# Coarse-Grained Descriptions of Dynamics for Networks with Both Intrinsic and Structural Heterogeneities

**DOI:** 10.3389/fncom.2017.00043

**Published:** 2017-06-12

**Authors:** Tom Bertalan, Yan Wu, Carlo Laing, C. William Gear, Ioannis G. Kevrekidis

**Affiliations:** ^1^Department of Chemical and Biological Engineering, Princeton UniversityPrinceton, NJ, United States; ^2^Department of Bioengineering, University of California, La JollaLa Jolla, CA, United States; ^3^Institute of Natural and Mathematical Sciences, Massey UniversityAuckland, New Zealand; ^4^Program in Applied and Computational Mathematics, Princeton UniversityPrinceton, NJ, United States

**Keywords:** networks heterogeneity, coarse-graining, polynomial chaos, projective integration

## Abstract

Finding accurate reduced descriptions for large, complex, dynamically evolving networks is a crucial enabler to their simulation, analysis, and ultimately design. Here, we propose and illustrate a systematic and powerful approach to obtaining good collective coarse-grained observables—variables successfully summarizing the detailed state of such networks. Finding such variables can naturally lead to successful reduced dynamic models for the networks. The main premise enabling our approach is the assumption that the behavior of a node in the network depends (after a short initial transient) on the *node identity*: a set of descriptors that quantify the node properties, whether intrinsic (e.g., parameters in the node evolution equations) or *structural* (imparted to the node by its connectivity in the particular network structure). The approach creates a natural link with modeling and “computational enabling technology” developed in the context of Uncertainty Quantification. In our case, however, we will not focus on ensembles of *different* realizations of a problem, each with parameters randomly selected from a distribution. We will instead study many *coupled heterogeneous* units, each characterized by randomly assigned (heterogeneous) parameter value(s). One could then coin the term *Heterogeneity Quantification* for this approach, which we illustrate through a model dynamic network consisting of coupled oscillators with one *intrinsic* heterogeneity (oscillator individual frequency) and one *structural* heterogeneity (oscillator degree in the undirected network). The computational implementation of the approach, its shortcomings and possible extensions are also discussed.

## 1. Introduction

Model reduction for dynamical systems has been an important research direction for decades; accurate reduced models are very useful, and often indispensable for the understanding, analysis, and ultimately for the design of large/complex dynamical systems. The relevant tools and techniques range from center manifold reduction close to bifurcation points (Guckenheimer and Holmes, 2002) to singular perturbation techniques [analytical (Bender and Orszag, 1999) or computational (Kevorkian and Cole, 1996)] and (more recently) to data-driven reduction methods [like PCA (Jolliffe, 2002), or non-linear manifold learning techniques (Coifman et al., 2005; Dsilva et al., 2016)]. While many such tools are well-established for ODEs and PDEs, the dynamics of networked dynamical systems (e.g., Newman et al., 2006) pose additional challenges.

We are interested here in large sets of dynamic units (agents, oscillators, cells) linked in a prescribed (and, for this paper, fixed) coupling pattern. Every unit consists here of a (relatively small) set of ordinary differential equations. The units are *intrinsically* heterogeneous, meaning that the parameters of this set of ODEs are sampled from a probability distribution. Once the overall system of ODEs modeling a large network is assembled, *any* generic dynamical system model reduction technique can be tried. For all-to-all coupled heterogeneous units, in particular, there has been extensive work taking advantage of the overall model structure, leading to the systematic reduction of such intrinsically heterogeneous assemblies (Ott and Antonsen, 2008, 2009; Laing, 2016).

Our illustrative example is a simulation of coupled phase oscillators whose dynamics are governed by the equations

(1) $$\begin{array}{l} \frac{\text{d}\varphi_{i}(t)}{\text{d}t}={\hat{\omega }}_{i}+\frac{K}{N}\sum_{j=1}^{N}A_{i,j}\sin(\varphi_{j}(t)-\varphi_{i}(t)), \end{array}$$

where *i* ∈ 1, …, *N*, and *A*_*i,j*_ ∈ {0, 1} is the adjacency matrix for a network with identical edges. This model was originally formulated by Yoshiki Kuramoto with all-to-all coupling (i.e., *A*_*i,j*_ = 1∀*i, j*) (Kuramoto, 1975, 1984). While we work with the simplified Kuramoto oscillator system, the methods presented here have been shown to work for more realistic coupled-oscillator systems as well, such as Hodgkin-Huxley-like neurons (Choi et al., 2016), metabolizing cells (Bold et al., 2007), gene-expression oscillations in circadian rhythms (ongoing work), or other candidate systems (Ashwin et al., 2016). In cases such as the Hodgkin-Huxley, where each unit is described by multiple dynamic variables, (e.g., membrane potential and gating variables), the analysis used here is repeated for each per-cell variable.

In order to construct a frame that moves with the average phase angle, we use states **θ** ∈ ℝ^*N*−1^, where

(2) $$\begin{array}{l} \theta_{i}(t)\overset{\text{def}}{=}\left(\varphi_{i}(t)-\frac{1}{N}\sum_{j=1}^{N}\varphi_{j}(t)\right),\;i=1,2,\ldots ,N-1, \end{array}$$

and $\theta_{N}\left(t\right)\overset{def}{=}-\overset{N-1}{\underset{j=1}{\sum }}\theta_{j}\left(t\right)$ (Hereafter, explicit time-dependence of θ(*t*) and φ(*t*) is usually not indicated, but can be assumed). Since the vector $\hat{\omega }$ of natural frequencies is not time-dependent, the transformation is particular to this problem and not really relevant to the model reduction technique discussed in this paper, though it ensures the existence of a steady **θ** state for sufficiently high values of *K*. This transformation is used to generate a new dynamical system

(3) $$\begin{array}{l} \frac{\text{d}\theta_{i}}{\text{d}t}={\hat{\omega }}_{i}-\frac{1}{N}\left[\sum_{j=1}^{N}{\hat{\omega }}_{j}\right]+\frac{K}{N}\left[\sum_{j=1}^{N}A_{i,j}\sin(\theta_{j}-\theta_{i})\right], \end{array}$$

∀*i* ∈ [1, *N*−1].

The main idea involves mathematical “technology transfer” from the field of *Uncertainty Quantification (UQ)* (Ghanem and Spanos, 2003; Xiu, 2010).

We assume that the long-time behavior of each unit in the assembly is characterized by (is a function of) its *identity*–the value(s) of the heterogeneous parameter(s). The problem can then be formulated as being distributed in (heterogeneous) parameter space (*p*-space) in a manner analogous to spatiotemporal processes “distributed” over physical space. The state θ_*i*_(*t*) for any unit *i* with identity *p*_*i*_ can be approximated in terms of appropriate basis functions not in physical space, but rather in “identity” space: heterogeneous parameter space.

(4) $$\begin{array}{l} \theta_{i}(t)=f(t;p_{i})\approx \sum_{k=1\ldots M}\alpha_{k}(t)\psi^{\left(k\right)}(p_{i}). \end{array}$$

The basis functions ψ^(*k*)^(*p*) are constructed as orthogonal polynomials in Section 3.2. Because, we are modeling cases in which the behavior of each unit is assumed to be a smooth function of identity (that is, nodes with similar identities are expected to behave similarly) a relatively short truncation of such a series may well be accurate enough if the right basis functions in *p*-space are chosen. In such a case, the number of ODEs to be solved reduces from the number of units O(*N*), to the number of terms in the series O(*M*). This approach, and its links to UQ modeling/computational developments (like the use of Smolyak grids for non-intrusive collocation-based simulation) has been explored in Laing et al. (2012b). Yet these developments were only applicable for all-to-all coupled, *intrinsically* heterogeneous assemblies of units.

The purpose of this paper is to generalize this approach by introducing a simple, yet non-trivial, extension. Namely, we consider *networks with non-trivial coupling structure*, i.e., not all-to-all coupled. In this work, all connections have the same strength; further extension to weighted connections is non-trivial, and the subject of current research. Each unit is now also characterized, beyond its ODE parameter values, by its *connectivity* in the network—the nature of its coupling with other units in the network, which in turn is quantified by features such as the unit's *degree* (its count of undirected connections). Different nodes have different connectivity features (imposed by the network structure)–we can therefore think of connectivity as *a type of heterogeneity* of our building block units: *structural* heterogeneity rather than *intrinsic* heterogeneity.

It should be noted that the steady state of similar dynamical systems have been predicted analytically (Ichinomiya, 2004; Restrepo et al., 2005). However, Ichinomiya (2004) considers the *N* → ∞ limit, whereas we deal with finite *N*. Additionally, Restrepo et al. (2005) requires that all the phases be known exactly to use a self-consistency argument. We are trying to obtain a reduced description, so necessarily we do not know (nor want to know) all the phases of all oscillators, but rather to approximate them.

In the system, Equation (1) to which we apply our coarse-graining strategy, we will see that the only connectivity feature that appreciably affects unit dynamics is the unit degree ${\hat{\kappa }}_{i}=\left(\overset{N}{\underset{j=1}{\sum }}A_{ij}\right)\in \left[0,N\right]$. That is, though we have previously shown that intrinsically similar nodes in this system (Moon et al., 2006) (and others Moon et al., 2015) have similar dynamics with all-to-all coupling, we show here that, with a non-trivial coupling topology network, nodes which additionally have the same (similar) degrees also have similar dynamics (possibly after a short initial transient). The degree of a node can be treated as another heterogeneous node parameter, whose probability distribution is the network degree distribution. We argue that the same approach which uses the distribution of an intrinsic heterogeneity, and led to the reduction of all-to-all unit assemblies in Moon et al. (2006) can be naturally extended to include a distribution over *structural heterogeneity* that leads to reduction of unit assemblies coupled in networks.

**Figure 1:**
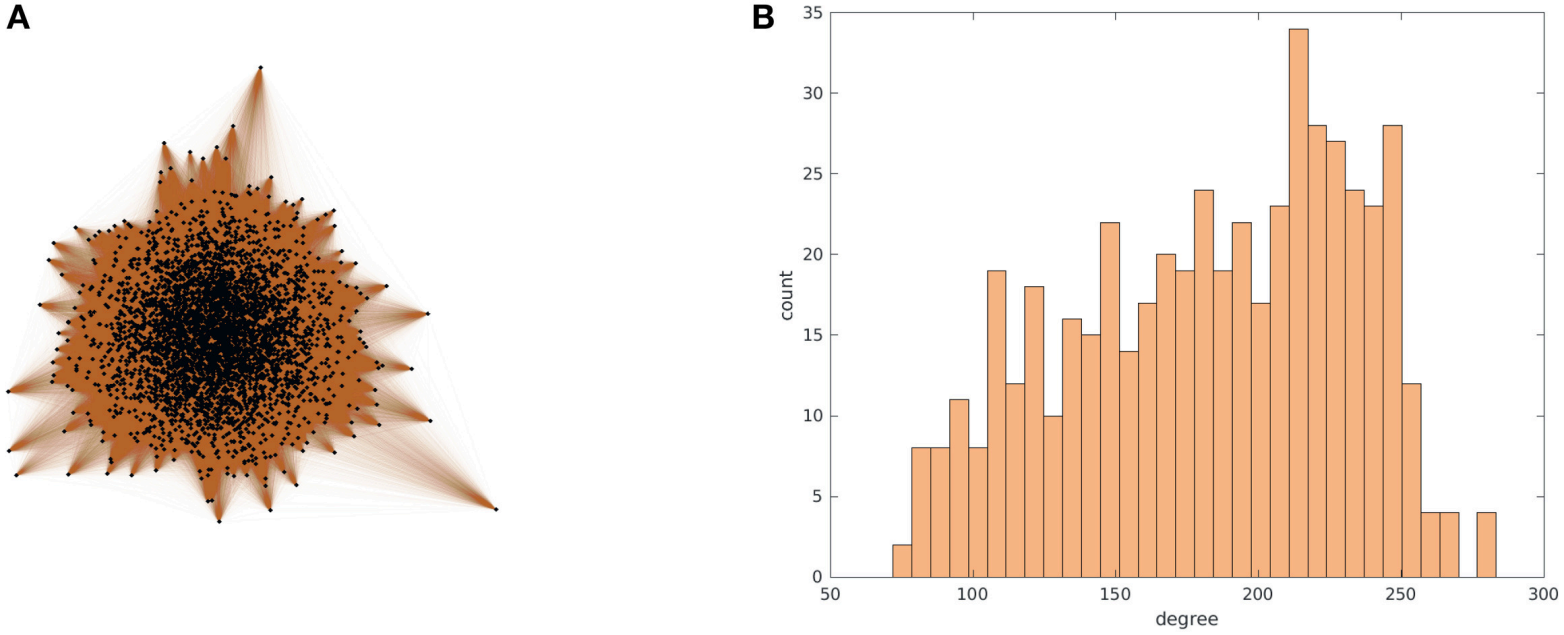
Visualization of a Chung-Lu network (Chung and Lu, 2002) with *N* = 4000 nodes, constructed using parameters *p* = 0.50, *q* = 0.90, and *r* = 0.50. In **(A)**, the network is plotted with MATLAB's 2D spectral projection-based layout. In **(B)**, the degree histogram is shown.

We demonstrate this in the simplest non-trivial representative setting we can put together: a set of coupled phase oscillators, characterized by heterogeneous frequencies ${\hat{\omega }}_{i}$ sampled from a prescribed distribution (here a truncated Gaussian)— but now *not* all-to-all coupled. Instead, the coupling *A* is in the form of a complex network, generated by a Chung-Lu process similar to that described in Laing et al. (2012a) and Chung and Lu (2002). The ideas presented here work also for general networks, if the degrees of the nodes are large enough; the Chung-Lu network is used as a convenient example. Likewise, the particular degree distribution tested here (shown in Figure 1) is not itself important, and networks with other degree distributions, such as power-law, or even non-monotonic degree distributions, can also be used. Here, we first generate a weight sequence *w*_*i*_, as

(5) $$\begin{array}{l} w_{i}=Np{\left(1-q(i-1)/N\right)}^{r}\;,\;i=1,2,\ldots ,N \end{array}$$

with parameters *p* = 0.50, *q* = 0.90, and *r* = 0.50. With **w**, we generate the connection probabilities *P* via

(6) $$\begin{array}{l} P_{ij}=P_{ji}=\min(\frac{w_{i}w_{j}}{\sum_{k}w_{k}},1). \end{array}$$

We concretely generate an adjacency matrix ***A*** from these probabilities by inverse transform sampling only above the diagonal of ***A***, and copying to the bottom triangle, to ensure that the network is undirected, with no self-loops.

Instead of following the behavior of each individual oscillator, we exploit the observation that similar oscillators have similar behavior and can be tracked together. For all to all coupling, “similar oscillators” is taken to imply similar natural frequencies, and we write the oscillator state as a function of natural frequency (Moon et al., 2006) and time. However, when a non-trivially structured coupling exists, “similar oscillators” implies not only intrinsic similarity but also *structural similarity*. In addition to the intrinsic explanatory parameter of the natural frequencies, the degree of each node appears to be an explanatory parameter which suffices (in our simple model) to capture the influence of the coupling structure on the behavior of each oscillator; yet other features such as in-degree or local clustering coefficient are also worth considering. If two oscillators have similar $\hat{\kappa }$-values and similar $\hat{\omega }$-values, then their time-dependent behavior is observed here to be similar, possibly after a short transient. Finding the relationship between oscillator characteristics (intrinsic and structural) and oscillator states generates a *coarse-grained* description, whereby the system state can be encoded in fewer independent variables.

We illustrate a number of coarse-grained modeling tasks facilitated by this reduction: accelerated simulation (via coarse projective integration), accelerated fixed point computation, continuation, and coarse-grained stability analysis [via time-stepper based coarse Newton-Krylov GMRES (Kelley, 1995, 2003) and Arnoldi algorithms (Saad, 2011)]. This creates a natural link between the reduction approach we present here, and our so-called equation-free framework for complex systems modeling Kevrekidis et al. (2003, 2004).

**Figure 2:**
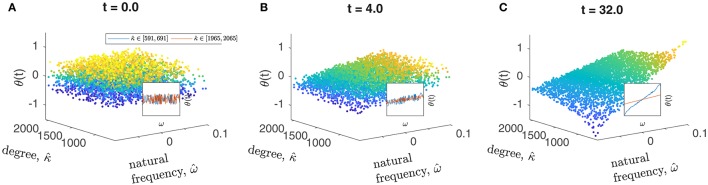
Oscillator states (phases) quickly slave to oscillator “identities.” The network oscillator states are initialized as a cloud in $\left[\hat{\omega },\hat{\kappa },\theta \left(t\right)\right]$ space, and are clearly seen to quickly rearrange onto a 2-D surface. Points are colored by θ-value. *N* = 4000 and *K* = 0.5 were used. Inset plots show slices at high and low $\hat{\kappa }$-values, including all oscillators in two bands of width 100. **(A–C)** show the same trajectory at three points in time.

In the end, what makes it all possible is the fundamental assumption about how heterogeneity (intrinsic as well as structural) affects the solution: “nearby” parameter values and “nearby” connectivities imply “nearby” dynamics. This is not always the case for *any* network, and so testing that this assumption holds must be performed on a case-by-case basis. For our networks, such a check is demonstrated in Figure 2. However, whether such a parameterization is possible is linked to the question of whether frequency-synchronization emerges, a subject for which an extensive literature exists (Dörfler and Bullo, 2014).

The remainder of the paper is organized as follows. First, we describe our illustrative example, a network of heterogeneous phase oscillators. We then give the form of our low-dimensional representation of the system state. We use our ability to transform back and forth between the two state representations to perform several computational tasks in the coarse-grained space, including the solution of initial value problems, the computation of fixed points, their stabilities, and bifurcations. Appendices include an analysis of the validity of using higher-order coarse-grained integration schemes.

## 2. An illustrative example of heterogeneous coupled oscillator networks

Our illustrative example is a network of coupled Kuramoto oscillators with heterogeneous natural frequencies ${\hat{\omega }}_{i}$, coupled in a stochastically generated network [here, a Chung-Lu network (Chung and Lu, 2002) with parameters *p* = 0.50, *q* = 0.90, and *r* = 0.50, an example instance of which is shown in Figure 1]. This type of model system was used in some previous reduction studies (Moon et al., 2006; Rajendran and Kevrekidis, 2011). The number of oscillators in the network is also a parameter we will vary; our base case is *N* = 196. A basic premise, which is corroborated by **Figure 6A**, is that the network is large enough (the number *N* of nodes is large enough) for the single realization to be representative of the expectation over all consistent network realizations. The *fine* dynamics are governed by the system of coupled ordinary differential equations (ODEs) (Equation 1), where the natural frequencies ${\hat{\omega }}_{i}$ and the node degrees (numbers of neighbors) ${\hat{\kappa }}_{i}$ are heterogeneous across the oscillators constituting the network. We remind the reader of our assumption that, of all structural node features that may affect the dynamics, it will be the node degree that matters here—so that the identity of node *i* is sufficiently described by its intrinsic parameter ${\hat{\omega }}_{i}$ and its structural parameter ${\hat{\kappa }}_{i}$. This assumption is supported first by Figure 2, and later, as we will see, more quantitatively by **Figure 6A**.

We further define rescaled versions of the two heterogeneous parameters, $x_{i}=({\hat{x}}_{i}-\text{mean}(\{{\hat{x}}_{j}\})/\text{stddev}(\{{\hat{x}}_{j}\})$ for *x* = ω, κ and *i, j* ∈ 1, …, *N*−1. These two transformations do not affect the fine dynamics of Equation (1) or (3); only the numerics of the implementation of the restriction *R* to a coarse-grained state representation, to be developed below. Without axis markings, Figure 2 for instance, would look the same whether ω × κ or $\hat{\omega }\times \hat{\kappa }$ were used for plotting.

The emergent functional dependence of the θ_*i*_ on the ω_*i*_ (the intrinsic heterogeneity only) was discussed in the all-to-all coupling context in Moon and Kevrekidis (2005) and Moon et al. (2006). There, we used a one-dimensional polynomial chaos expansion (PCE) to describe the reduced problem for *A*_*ij*_ = 1∀*i, j* ∈ [1, *N*], *i* ≠ *j*, so that all nodes have degree ${\hat{\kappa }}_{i}=N-1$. In this paper, we again expect the oscillator states to quickly become smooth (and time-dependent!) functions of their identities, but node *i*'s identity now includes *both* ${\hat{\omega }}_{i}$ and ${\hat{\kappa }}_{i}$. For *K* sufficiently large so that a steady state of Equation (1) exists, we indeed observe that the states of randomly initialized oscillators quickly approach an apparently smooth surface in ω × κ space (see Figure 2) suggesting that a low-order series truncation of the type described in Equation (8) may constitute a good description. This motivates the use of a functional fit of the coefficients [the few α_*k*_(*t*) in Equation 8] to the data [the many θ_*i*_(*t*)] as a coarse representation.

In previous work (Rajendran and Kevrekidis, 2011), we have used a projection onto the eigenvectors of the discrete Laplacian on the graph to describe the dependence of oscillator state on structural heterogeneity, while using a one-term/linear fit to account for dependence on intrinsic frequency.

## 3. Low-dimensional representation

### 3.1. Polynomial chaos

Given our observation that oscillator behavior quickly becomes a function of oscillator *identity*, we want to describe the long-term dynamics of the oscillator phase angles as a smooth function θ(*t*; ω, κ). The phase angle of the *i*-th oscillator is then given by θ_*i*_(*t*)≡θ(*t*; ω_*i*_, κ_*i*_). Since our two heterogeneities (the intrinsic and the structural) are here independent, the basis functions are a tensor product of two independent polynomial bases

(7) $$\begin{array}{l} \psi^{\left(\gamma \right)}(\omega ,\kappa )=\psi_{\omega }^{\left(\gamma_{k,\omega }\right)}(\omega )\psi_{\kappa }^{\left(\gamma_{k,\kappa }\right)}(\kappa ). \end{array}$$

This is a special case; the formulation will still in principle be applicable for parameters with correlated joint probability distributions if one constructs an appropriate set of basis functions (Navarro et al., 2014; Deck, 2015).

We now express the network dynamics in the form of a series expansion in a truncation of this tensor product basis as

(8) $$\begin{array}{l} \theta (t;\omega ,\kappa )\approx \sum_{k=1}^{M}\alpha_{k}(t)\psi^{\left(k\right)}(\omega ,\kappa )\equiv \sum_{k=1}^{M}\alpha_{k}(t)\xi^{\left(\gamma_{k,\omega }\right)}(\omega )\zeta^{\left(\gamma_{k,\kappa }\right)}(\kappa )\\ \;G=\{\gamma_{k}=(\gamma_{k,\omega },\gamma_{k,\kappa }):0\leq \gamma_{k,\omega },\gamma_{k,\kappa }\in \mathbb{Z},\;\gamma_{k,\omega }+\gamma_{k,\kappa }\\ \;\leq p_{\text{max}}\},\\ \;M=||G||=(1+p_{\text{max}})(2+p_{\text{max}})/2 \end{array}$$

where the α_*k*_(*t*) are time-dependent coefficients, $\xi^{\left(\gamma_{k,\omega }\right)}\left(\omega \right)$ are basis functions arising from the intrinsic heterogeneity dependence and $\zeta^{\left(\gamma_{k,\kappa }\right)}\left(\kappa \right)$ are basis functions arising from the structural heterogeneity dependence. Within the truncation of the set of functions *G* included in the basis, the ordering of the basis can be chosen arbitrarily, and so we substitute the vector index **γ**_*k*_ = (γ_*k*, ω_, γ_*k*, κ_) with a scalar index 1 ≤ *k* ≤ *M*.

The analogy with UQ now manifests itself in our choice of the two independent basis sets: each one of them is chosen to be a polynomial chaos basis in the corresponding heterogeneous (in analogy to random) parameter. Each set of polynomials is orthogonal with respect to the probability density of the corresponding heterogeneous parameter, and the joint heterogeneity probability density is just the product of the two unidimensional, independent heterogeneity probability densities, so that Equation (7) is satisfied, as shown in **Appendix A.1**.

Note that in Equation (8), we specify that γ_*k*, ω_+γ_*k*, κ_ ≤ *p*_*max*_. This allows us to say that our two-dimensional polynomials are of *total degree* ≤ *p*_max_. An alternative truncation rule would be to require that γ_*k*, ω_ ≤ *p*_*max*_ and γ_*k*, κ_ ≤ *p*_*max*_ (or even to place separate bounds on γ_*k*, ω_ and γ_*k*, κ_, allowing for some anisotropy in the details, a topic for separate investigation). Both approaches can be found in the literature.

This allows, per Equation (8), an approximation of the behavior as a time-dependent two-dimensional surface in one intrinsic dimension [here, the (normalized) natural frequencies ω], and one structural dimension [here, the (normalized) node degrees κ]. We repeat that this tensor product basis, limited to those polynomials of total order less than some desired maximum, is a truncated orthogonal basis for the 2D space weighted by probability densities that are products of two marginal distributions. Some standard distributions and their corresponding families of orthogonal polynomials (Xiu, 2010) are given in Table 1.

**Table 1:** Frequently encountered probability distributions and the corresponding weighted orthogonal polynomial families.

**Distribution**	**Polynomials**
**DISCRETE**	
Binomial	Kravchuk
Poisson	Charlier
Negative binomial	Meixner
Hypergeometric	Hahn
**CONTINUOUS**	
Gaussian	Hermite
Gamma	Laguerre
Beta	Jacobi
Uniform	Legendre

In classical, Galerkin methods an inner product is taken between the governing evolution equations and each basis function, producing ODEs for the dynamics of the expansion coefficients [the α_*k*_(*t*) in Equation 8] by exploiting orthogonality. A similar approach could be taken here (through analytical computation of the inner product integrals if possible, else through numerical quadrature). Instead, we do not directly calculate the temporal rates-of-change of the expansion coefficients, but infer features of the coefficient dynamics from brief bursts of simulation of the dynamics of the full system (see Section 4). This equation-free approach relies on our ability to go back and forth between fine descriptions of the system state (the θ_*i*_ values), and coarse ones (the α_*k*_ values). This is analogous to a non-intrusive (black-box, input-output) approach to using polynomial chaos in UQ.

### 3.2. Equation-free numerics

The choice of the polynomial basis sets follows the selection of the appropriate heterogeneity distribution. For several frequently encountered distributions, the bases have been tabulated (e.g., Table 1) from original generalized polynomial chaos references (see e.g., Xiu, 2010). If not already available in such tables, one can construct the basis polynomials e.g., through Gram-Schmidt orthogonalization with inner products in the space weighted by the distribution function. A couple of non-trivial considerations arising in our case are that (a) our structural heterogeneity parameter (the node degree) takes integer values, and so the degree distribution is discrete; and (b) often we may encounter problems for which the heterogeneity distribution is not explicitly known, but has to be estimated from specific system realizations (so, from large enough samples). For the case of *explicitly unknown* but *sampled* distributions (whether discrete or continuous) we have used here the moments of the sampling of the heterogeneity parameters for our particular network realization to extract the corresponding polynomials (using SVD-based pseudo-inverses) (Oladyshkin and Nowak, 2012).

**Figure 3:**
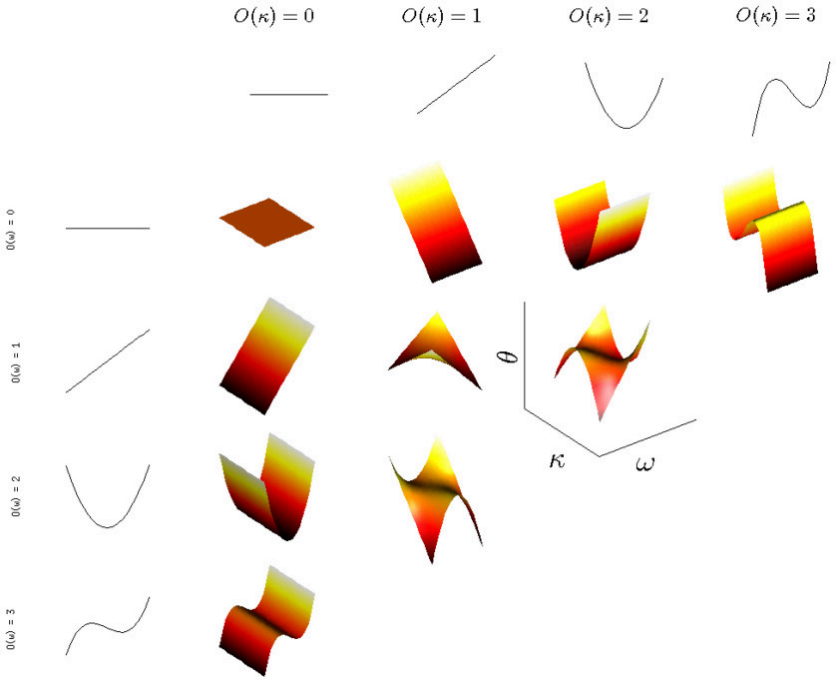
2D polynomials (orthogonal with respect to the ω, κ density described in the text) are generated for a maximum total polynomial order of *p*_max_ = 3.

Results of the moment-based polynomial generation method, which we used to generate all 1D polynomials used in this paper, are shown in Figure 3. Here, the marginal samplings of degrees and natural frequencies were used separately to generate 1D polynomials via moments, and then a 2D basis was defined from the tensor product of these 1D bases, with the restriction O(ω)+O(κ) ≤ *p*_max_ placed on the total polynomial degree of the 2D basis functions used.

For the expansion in Equation (8), we used a set of 2D basis functions. Once the relevant polynomials have been constructed, the appropriately defined inner product also allows us (whether for continuous or for discrete distributions, explicitly known or not) to find the coefficients α_*k*_ in Equation (8) for a given observation θ(*t*) of the system states for a particular system realization (sampling of the distribution). This can be accomplished directly (via numerical approximations of the relevant inner products), using the orthogonality of the ψ^(*k*)^,

(9) $$\begin{array}{l} \alpha_{k}=\frac{\int_{D}f(x)\psi^{\left(k\right)}(x)\text{d}\Gamma (x)}{\int_{D}{\left(\psi^{\left(k\right)}(x)\right)}^{2}\text{d}\Gamma (x),} \end{array}$$

where we use the Lebesgue integral $\int_{D}g\left(x\right)d\Gamma \left(x\right)=𝔼\left[g\left(x\right)\right]$. For our problem, with one continuous and one discrete variable, this can be written concretely as $\int_{D}g\left(x\right)d\Gamma \left(x\right)=\int \overset{N}{\underset{\kappa =0}{\sum }}g\left(\kappa ,\omega \right)\rho_{\kappa }\left(\kappa \right)\rho_{\omega }\left(\omega \right)d\omega$, (ω, κ) ∈ *D*, where ρ_κ_ is the (discrete) probability mass function for the degrees, and ρ_ω_ is the (continuous) probability distribution function for the natural frequencies. In separate work (Choi et al., 2016), we examine the computation of this integral for the case when the problem can be recast as a PDE, and so the coupling sum in Equation (1) and (3) can also be written as an Lebesgue integral. There, we consider standard Monte Carlo integration in addition to Gaussian quadrature and a repurposing of anchored ANOVA. These latter methods have the benefit of allowing integrals to be computed using only a few key virtual oscillators, with anchored ANOVA having the additional benefit of decreased scaling sensitivity to the number of random dimensions (two in this paper). However, these benefits require that the original model be recast as PDEs continuous in both time and the random dimensions.

Here, we take the alternate approach of finding the α_*k*_ indirectly through least squares fitting, minimizing the squared residual norm σ with respect to the coefficients α_*k*_ (here using a QR algorithm).

(10) $$\begin{array}{l} \sigma =||f(x)-\overset{M}{\underset{k=1}{\sum }}a_{k}\psi^{\left(k\right)}(x)|{|}_{2}^{2}\\ \;\approx \hat{\sigma }=\sum_{i=1}^{n_{\text{samp}}}w(x_{i}){\left(f(x_{i})-\sum_{k=1}^{M}\alpha_{k}\psi^{\left(k\right)}(x_{i})\right)}^{2}, \end{array}$$

where $\underset{n_{\text{samp}}\to \infty }{\lim}\hat{\sigma }=\sigma$ according to the law of large numbers, and the weights *w*(***x***_*i*_) are still to be decided.

For a (large) finite sample ***x***_*i*_, *i* = 1, …, *n*_samp_, if we take the partial derivative $\partial \hat{\sigma }/\partial \alpha_{k}$, and use the fact that $\overset{n_{\text{samp}}}{\underset{i=1}{\sum }}w\left(x_{i}\right)\psi^{\left(l\right)}\left(x_{i}\right)\psi^{\left(k\right)}\left(x_{i}\right)=0$ if *l* ≠ *k* (orthogonality) to remove some terms, then we find that

(11) $$\begin{array}{l} \alpha_{k}=\frac{\sum_{i=1}^{n_{\text{samp}}}w(x_{i})f(x_{i})\psi^{\left(k\right)}(x_{i})}{\sum_{i=1}^{n_{\text{samp}}}w(x_{i}){\left(\psi^{\left(k\right)}(x_{i})\right)}^{2}}. \end{array}$$

So, as long as we accept that

(12) $$\begin{array}{l} \sum_{i=1}^{n_{\text{samp}}}w(x_{i})g(x_{i}) \end{array}$$

is a good approximation to

(13) $$\begin{array}{l} \int_{D}g(x)\text{d}\Gamma (x), \end{array}$$

we obtain the same formulas for the α_*k*_.

Suppose ***x*** has a density ρ(***x*** ∈ *D*), so Equation (13) can be written as

(14) $$\begin{array}{l} \int_{D}g(x)\rho (x)\text{d}x, \end{array}$$

If the ***x***_*i*_ are chosen randomly in accordance with ρ(***x***), then Equation (12), where *w*(***x***_*i*_) = 1/*n*_samp_, is a good approximation to Equation (14). This is just Monte Carlo integration, and the law of large numbers gives

(15) $$\begin{array}{l} \underset{n_{\text{samp}}\to \infty }{\lim}\frac{1}{n_{\text{samp}}}\sum_{i=1}^{n_{\text{samp}}}g(x_{i})=\int_{D}g(x)\rho (x)\text{d}x, \end{array}$$

## 4. Coarse computational modeling tasks

Beyond their conceptual simplification value, collective (coarse) variables can be valuable in facilitating the computer-assisted study of complex dynamical systems by accelerating tasks such as direct simulation, continuation, stability, and bifurcation analysis for different types of solutions. To accomplish this acceleration, the equation-free approach (Kevrekidis et al., 2003, 2004) is predicated on the ability to map between corresponding fine and coarse descriptions of the same system.

This is accomplished through the definition of a *restriction operator* *R*:ℝ^*N*−1^ → ℝ^*M*^ which maps from fine states **θ**(*t*) to corresponding coarse states **α**(*t*) by minimizing the residual σ(**α**(*t*)) from Equation (10). We also need to define the counterpart of restriction: a *lifting operator* *L*:ℝ^*M*^ → ℝ^*N*−1^ which maps **α** vectors to **θ** vectors by setting the θ_*i*_ values equal to the right-hand-side of the approximant in Equation (8) evaluated at the corresponding (ω_*i*_, κ_*i*_).

One more important thing to note before proceeding to demonstrating the approach is that the lifting operator is, in general, a one-to-many relation; there are many fine realizations of the process that are mapped to the same coarse representation—coarse-graining (e.g., averaging) loses information. If the problem can be usefully coarse-grained, any of these consistent fine realizations, or the average of several of them, can be used practically in the definition of the coarse time-stepper below; we may think of the coarse-time-stepper as the *expected value* over all such consistent realizations. In singularly, perturbed multiscale problems one can clearly see how the memory of the details of the lifting are quickly forgotten, suggesting that any consistent fine realization is “good enough” to estimate this expectation (Gear et al., 2002; Kevrekidis and Samaey, 2009).

The *L* and *R* operators combine to define a coarse timestepper Φ_τ, *C*_, in

(16) $$\begin{array}{l} \;\Phi_{\tau ,C}:{\mathbb{R}}^{M}\to {\mathbb{R}}^{M}\\ \;\Phi_{\tau ,F}:{\mathbb{R}}^{N-1}\to {\mathbb{R}}^{N-1}\\ \;\Phi_{\tau ,F}[\theta (t)]=\theta (t+\tau )\\ \;=\int_{s=t}^{s=t+\tau }\frac{\text{d}\theta (s)}{\text{d}t}\text{d}s\\ \Phi_{\tau ,C}[\alpha (t)]=\alpha (t+\tau )\\ \;\equiv (R\circ \Phi_{\tau ,F}\circ L)[\alpha (t)] \end{array}$$

This is the timestepper for the (unavailable) coarse-grained dynamical system, approximated through observing the results of short bursts of appropriately initialized fine-grained simulations. A single evaluation of this coarse time-stepper, by itself, does not provide any computational savings; it is the way we design, and process the results of, several such coarse time-steps that leads to computational benefits. Using traditional numerical analysis codes (initial value solvers, fixed point solvers) as templates for wrapper codes around the coarse timestepper, tasks like accelerated simulation, coarse-grained stability and bifurcation analysis, optimization, and controller design, can be performed. This wrapper technology is described in detail (and fruitfully used to explore model coarse-graining across disciplines) in a series of publications (Theodoropoulos et al., 2000; Kevrekidis and Samaey, 2010). What is important here is not the established wrapper algorithms technology; it is the selection of coarse observables, leading to the appropriate definition of the coarse time-stepper, that makes the entire program feasible and useful.

### 4.1. Coarse initial value problems

We can use the coarse timestepper to accelerate the computation of dynamic trajectories of the system, through Coarse Projective Integration (CPI) (Gear and Kevrekidis, 2003; Lee and Gear, 2007). Given a coarse initial condition **α**(*t* = 0) we lift to a consistent fine scale state *L*[**α**(*t* = 0)] and use it to initialize a fine scale numerical integrator. We run for a short time τ (the inner step) and we record the final coarse state by restricting the corresponding fine state, *R*[**θ**(*t*+τ)]. We use these two coarse states to estimate the coarse time derivative, which we then use in the forward Euler formula to project forward in time the coarse state for a (large, coarse) time step *h* (the outer step). This constitutes the simplest coarse projective forward Euler integration scheme:

(17) $$\begin{array}{l} \alpha (t+h)=\alpha (t)+h\frac{\Phi_{\tau ,C}[\alpha (t)]-\alpha (t)}{\tau } \end{array}$$

A slightly more sophisticated approach would also take two points separated by an inner step size τ to approximate the rate-of-change of **α**, but only after first performing a healing integration in the fine equations (Gear et al., 2002). If the lifted representation of the state **α**(*t*), as projected from the previous timestep **α**(*t*−*h*), is slightly off the hypothetical slow manifold in the fine space, this short healing trajectory dampens the fast components which are not captured in the coarse representation (Gear et al., 2005; Vandekerckhove et al., 2011; Antonios et al., 2012).

It is important to note here that in all our CPI computations we used a single network (with a single ω vector) to generate the polynomial basis functions *and* to lift to at every projective step. This can be thought of as a “single instance” CPI; one may also consider CPI for the expected behavior over all networks that share the same degree as well as ω distributions—in which case one should lift to many consistent network realizations and average over them. This issue will be examined more closely below.

**Figure 4:**
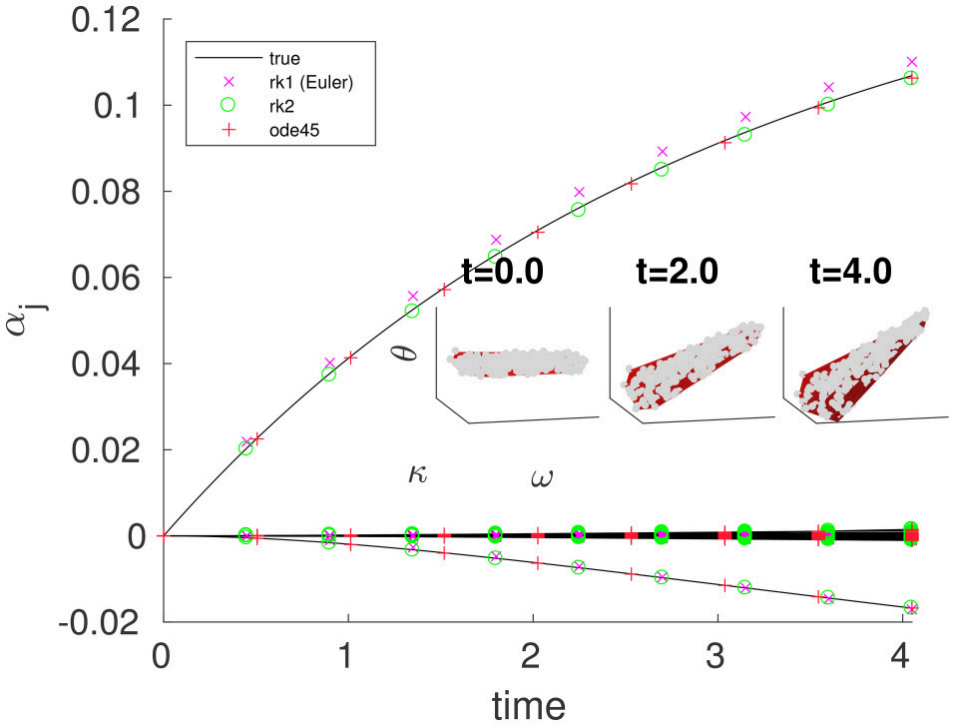
Coarse projective integration shows smooth evolution of the first few leading PCE coefficients α_*j*_, with some corresponding fine states visible in the 3D insets. Black curves (in the main figure) and dense gray scatters (in the insets) were obtained by full fine integration with the same initial θ conditions using MATLAB's ode23. Colored points (in the main figure) and red surfaces (in the insets) were obtained via CPI, using several different integrators. At each coarse step, $\frac{d\alpha_{k}}{dt}$ was estimated ∀*k* = 1, …, *M* (where *M* = 28) by drawing *M* chords through the restrictions of the last two points in a brief burst of fine integration of τ = 0.05 time units. At the times indicated, we make inset plots with red surfaces corresponding to the lifted CPI state and gray scatters corresponding to the closest (in time) state in the true trajectory. These should be compared to Figure 2. We performed the same task for several outer integrators: two explicit Runge-Kutta integration schemes, and a coarse wrapper around the built-in MATLAB integrator ode45 are compared to the restrictions of points in the fine trajectory starting from the same lifted initial condition. For the two explicit Runge-Kutta integrators, an outer step of *h* = 0.45 was used. For ode45, an absolute tolerance of 10^−6.0^ and a relative tolerance of 10^−12.0^ were used. *N* = 300, *K* = 1, and *M* = 28 were used. The ω-values were drawn from a truncated normal distribution supported on [−0.100, 0.100], with zero mean and standard deviation 0.060.

**Figure 5:**
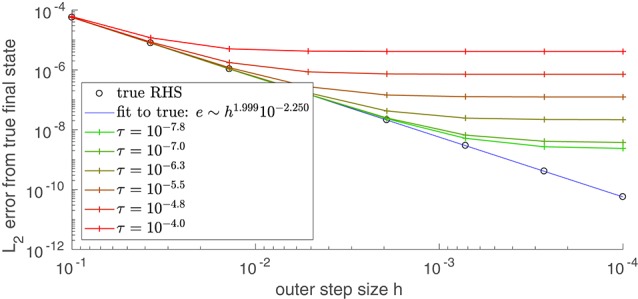
As the inner step size τ decreases, the error (compared to direct integration) of a projective (not coarse-projective) integration becomes bounded by the integrator's intrinsic (outer) step size *h*. The true solution at *t* = 0.417 was found by integrating using MATLAB's ode45 with an absolute tolerance of 10^−12.0^ and a relative tolerance of 10^−12.0^ The series of black circles give the error at *t* = 0.417 that results from using integration using the true RHS function (Equation 3) in an explicit second-order Runge-Kutta integration scheme of (outer) step size *h*. The colored curves use the same integrator and outer step size, but approximate the RHS function with the difference map *f*_τ_(θ(*t*)) = θ(*t*)−Φ_τ, *F*_[θ(*t*)], analogous to the coarse difference map of Equation (18). Error was evaluated by taking the norm of the vector difference between the projective integration solution **θ**(0.417) and the true solution. Compare this to Figure A1 in the Appendix, in which a similar analysis is performed on a system of two ODEs modeling a single reversible reaction.

Results of applying the simpler scheme to the coupled oscillator problem with coarse variables obtained by a 2*D* PCE fit are shown in Figure 4. In general, the coarse time-stepper results can be used to estimate the coarse right-hand-side function $\dot{\alpha }$, which is not available in closed form. On demand numerical estimates of this right-hand-side through short bursts of appropriately initialized fine simulation allow us to use other existing integrators, such as MATLAB's ode45, to approximate computationally coarse trajectories (also shown in Figure 4). **Appendix B.1** contains a quick illustration of the useful properties of such projective initial value solvers. It is shown there that, under reasonable conditions, the order of a *projective* integrator templated on a two-step Runge-Kutta initial value solver (including the additional estimation step for the coarse time derivatives) is the same as the order of the *actual* Runge-Kutta initial value solver. Figure 5) confirms this for projective integration of the fine equations for our model.

This approximation of the coarse right-hand-side function can be used for other computational tasks besides the computation of dynamic trajectories.

### 4.2. Coarse fixed point computation

The coarse time-stepper can be used to define a coarse difference

(18) $$\begin{array}{l} F_{\tau }[\alpha (t)]=\Phi_{\tau ,C}[\alpha (t))]-\alpha (t). \end{array}$$

Steady states of the fine time-stepper are clearly zeroes of this difference; one expects that the zeroes of the coarse difference correspond to coarse steady states of the original problem. *F*_τ_ can therefore be used to find coarse steady states involving only *M* variables. Iterative, matrix-free linear algebra lends itself to finding zeroes of such a problem in the absence of explicit equations for the dynamics of the coarse variables α_*j*_, We used a Krylov-type matrix free technique (Newton-Krylov GMRES) to converge to such coarse steady states.

**Figure 6:**
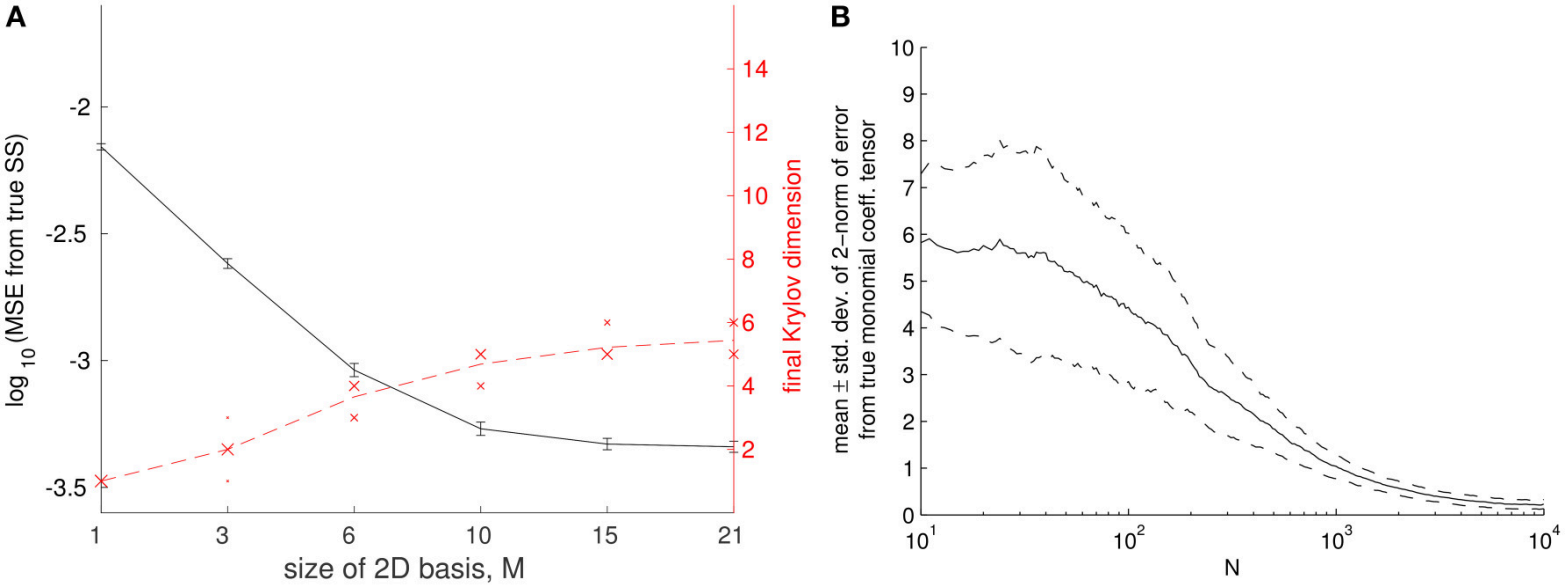
(A) Coarse fixed point computations, and their sensitivity to the size of the coarse basis used. Computations were repeated for different samples of network and natural frequencies and the error bars are indicative of the resulting variation in the solution. Error was computed between lifted fixed points of Equation (18) (with τ = 0.05), and steady states of Equation (3). The norm used was the mean squared error (MSE) across the *N*−1 nodes. Thirty-two replicates were used per value of the independent variable, an absolute tolerance of 10^−6.0^ was used for the outer Newton solver, error bars are ± 1 standard deviation, and *K* = 1 was used. **(B)** Convergence of the orthogonal polynomials based on (increasingly larger) finite networks. As *N* increased, the polynomials generated via sample moments approached the *N*=10,000 polynomials. Error was quantified in the 2-norm of the monomial coefficient tensor *C* in $\psi^{\left(k\right)}\left(\omega ,\kappa \right)=\left(\overset{p_{k,\omega }}{\underset{l=0}{\sum }}C_{\omega ,k,l}x^{l}\right)\left(\overset{p_{k,\kappa }}{\underset{l=0}{\sum }}C_{\kappa ,k,l}x^{l}\right)$, where the pair *p*_*k*_ gives the orders of the two one-dimensional polynomials.

A Newton-Krylov iteration to find such a state is depicted in Figure 6. In Newton-Krylov GMRES (Generalized Minimal RESidual), the inner linear problem of an outer (non-linear) Newton-type solver is solved by GMRES, in which the solution to ***Bx*** = ***b*** is assembled in a space derived from the *nth* Krylov subspace ${\left\{B^{j}r_{0}\right\}}_{j=0,\ldots ,n-1}$, where *r*_0_ is the residual of the initial iterate (and ***B***, which is not computed, is the Jacobian of Equation 18).

Here, we work again with *N* = 196 node networks. The basis polynomials are computed from *a single* network realization (a large, 10, 000 node Chung-Lu network); because the support of the degree distributions for a *N* = 10,000 and for a *N* = 196 network are not the same, the degrees and frequencies are normalized as described in Section 2. However, now we construct 32 realizations of networks consistent with the chosen degree distribution, and perform our fixed point computation for each one of them. We do *not* regenerate polynomials for each of these realizations; we observe them on the “large sample” polynomials; this is justified in Figure 6B, where we approach the *N* = 10, 000 polynomials as *N* increases, by generating with sample moments. The effect of regenerating polynomials within a larger computation is considered further in Rajendran et al. (2012, 2016).

We report the sensitivity of the results to the basis size *M* in Figure 6A, where the error bars are indicative of the variation across our 32 network samples. We can also see in Figure 6A that the dimension of the Krylov subspace (at the final Newton iteration before convergence) initially closely follows the size of the 2D basis we use (it uses “all of *M*”) but later on plateaus.

### 4.3. Coarse stability computations: eigenvalues and eigenvectors

(Coarse) eigenvalues of the Jacobian of the (coarse) difference map (Equation 18) upon convergence to its (coarse) fixed points can be used to establish the stability of these fixed points and help determine the nature of their potential (coarse) bifurcations. These eigenvalues μ_*i*_ are related by

(19) $$\begin{array}{l} \lambda_{i}\approx {\hat{\lambda }}_{i}=\ln(\mu_{i}+1)/\tau \end{array}$$

to the corresponding eigenvalues ${\hat{\lambda }}_{i}$ of the Jacobian of the (unavailable) coarse differential evolution equations; in turn, these should coincide with the leading eigenvalues λ_*i*_ of the actual problem (the leading eigenvalues of the *fine* differential equations). For *M* > 3 coarse eigenvalues μ_*i*_ were obtained through the Jacobian-free implicitly restarted Arnoldi Method (IRAM) (Kelley, 1995; Lehoucq et al., 1998) applied to the coarse difference operator (Equation 18). For *M* ≤ 3, a forward finite-difference Jacobian with a fixed step size of 0.001 was computed and the eigenpairs calculated directly with the QZ algorithm implemented in MATLAB's eig.

**Figure 7:**
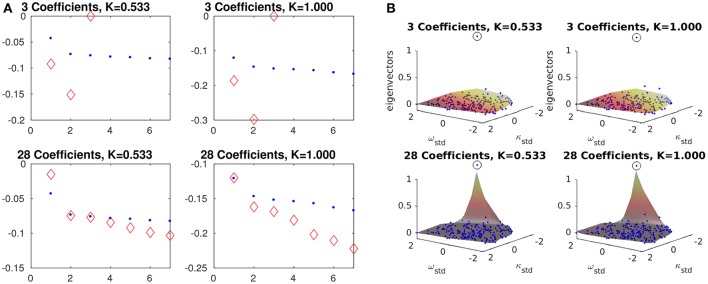
Comparison of coarse and fine eigencomputations at different *K*-values and basis sizes *M*. **Left** and **right** columns in each subfigure, respectively, are for values of K close to the main SNIPER bifurcation point and far away from it (see text and Figure 8). **Top** and **bottom** rows, respectively, are for small and large basis sizes. Eigenpairs obtained from Equation (18) (with τ = 0.05, *N* = 196, and *M* = 28) are similar to those obtained from Equation (3). True eigenvalues and eigenfunctions (small blue points) were obtained using MATLAB's eig on an analytical Jacobian of Equation (3). Approximate eigenvalues (large red diamonds), obtained via implicitly restarted Arnoldi iteration (IRAM) with the transformation of Equation (19), converge to the the fine eigenvalues λ_*i*_ as *M*, the number of α_*k*_ coefficients, rises. For larger values of *K*, the eigenvalues are all increasingly negative, though the ratio between the first and second eigenvalue (about 0.6) does not change by much. In **(A)**, the horizontal axis of each plot is an index across eigenvalues, while the vertical axis is eigenvalue. In **(B)**, the lifted view of the leading coarse eigenvector visually approaches the leading fine eigenvector. The coarse eigenvector was evaluated as a surface (in a manner similar to Equation 8) at a fine grid of points within the convex hull of the sampled (ω, κ) points. The “eigensurface” corresponding to the slowest eigenvalue appears to approach an indicator function on the oscillator whose extreme (ω, κ) pair makes it the most susceptible to “desynchronization” with decreasing *K*. Eigenpairs were chosen to match the right (synchronized) inset plot in Figure 8, and the point closest to the turning point along the branch of coarse fixed points in that figure.

As the number of coarse variables is increased, and therefore the quality of the coarse approximation improves, one expects these coarse eigenvalue estimates to approach the leading eigenvalues of the analytical fine Jacobian of Equation (3), located through any eigensolver. In Figure 7A, we demonstrate this convergence of the approximate eigenvalues ${\hat{\lambda }}_{i}$ to the leading fine eigenvalues λ_*i*_ with increasing *M*. Figure 7B shows the corresponding convergence of a coarse eigenvector (the one corresponding to the smallest absolute value of ${\hat{\lambda }}_{i}$) to the fine eigenvector corresponding to smallest absolute value of λ_*i*_. Note that lifting is necessary to make a comparison between θ and α eigenvectors. On the other hand, the transformation (Equation 19) is necessary not because of our coarse and fine spaces, but because the μ eigenvalues come from the Jacobian of a finite-time flow map while the λ eigenvalues come from the Jacobian of a vector of differential equations.

In performing computations involving finite differences, we used a value of $\sqrt{\text{machine precision}}$ (according to Press et al., 1992), which is ~10^−7^ for IEEE standard double-precision floating point variables in MATLAB.

### 4.4. Coarse continuation/bifurcation diagrams

**Figure 8:**
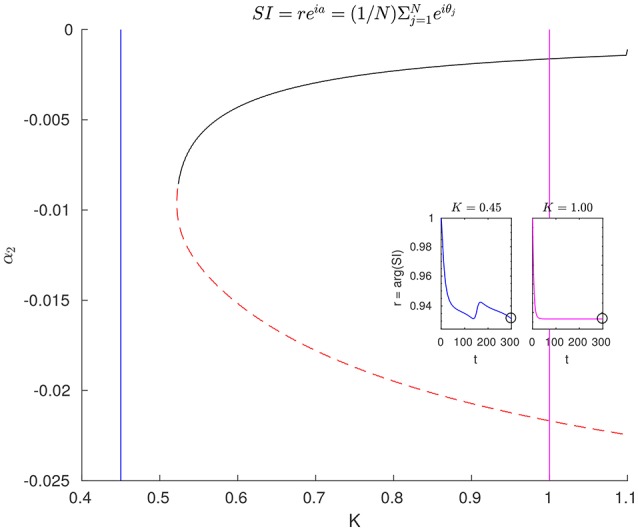
Bifurcation diagram, coarse, and fine. Coarse fixed-point solutions can be used to generate a bifurcation diagram in the parameter *K*, via pseudo-arclength continuation of fixed points of the coarse flow map in Equation (16) (with τ = 0.30, *N* = 196, and *M* = 28). At the point where the color of the curve changes from red to black, one eigenvalue passes through zero. This marks a change from a stable to an unstable branch. The two inset plots show representative trajectories of the real magnitude *r* of the complex synchronization index $re^{ia}=\frac{1}{N}\overset{N}{\underset{j=1}{\sum }}e^{i\theta_{j}}$. This quantity can be thought as a vector pointing to the mean phase angle, whose length approaches 1 as the oscillators approach perfect (phase-)synchronization. In the right half of the bifurcation diagram, trajectories approach stable steady states, where oscillators are completely (frequency-)synchronized. In the left half, one (or more) rogue oscillator(s) travel(s) around the phase ring alone, slowing briefly when passing through the cluster of synchronized oscillators. For both insets, the initial condition was θ_*i*_ = 0∀*i*.

To build a coarse bifurcation diagram (See Figure 8), we performed pseudo-arclength continuation (Keller, 1977; Kelley, 2005) for the coarse fixed points. We computed branches of coarse solutions to 0 = *F*_τ_(**α**; *K*) as the global parameter *K* is varied. To trace out these solution branches, steps were taken in (pseudo-)arclength along the branch rather than in *K*. This allows the continuation to extend naturally beyond turning points. At some point along this continued branch of solutions, one of the computed eigenvalues becomes positive. At this point, the line color is changed to indicate that the new branch comprises unstable solutions.

Beyond these branches (to the left) we know that a limit cycle solution arises: a periodic orbit characterized by one free *rogue oscillator*, which performs full rotations and only momentarily slows down as it passes through the remaining pack of clustered oscillators (Moon and Kevrekidis, 2005). In dynamical systems terminology this is a “SNIPER” (saddle-node infinite period) bifurcation (Strogatz, 2001). The insets in Figure 8 show transient dynamics in terms of the *synchronization index* *r* in $re^{ia}=\frac{1}{N}\overset{N}{\underset{j=1}{\sum }}e^{i\theta_{j}}$ (the real magnitude of the complex Kuramoto order parameter; see e.g., Skardal et al., 2011). The presence of this rogue oscillator means that the coarse representation of Equation (8) is not particularly accurate/informative to the left of *K*_*c*_, without explicitly including the rogue's value of θin the set of coarse variables, as was done, for example, in Moon et al. (2006).

## 5. Discussion

In this paper, we have demonstrated that a general network of coupled, intrinsically heterogeneous oscillators can be usefully described using a small number of collective dynamic variables. These variables are the time-dependent coefficients of an expansion of the complete state of the network in terms of a set of orthogonal polynomials. The polynomials are products of univariate polynomials in the parameters describing the *intrinsic heterogeneity* of a given oscillator, and a *structural* heterogeneous property (here, the degree) indicative of the connectivity of the oscillators in the network. Our results extend previous work which only considered all-to-all coupled networks, in which the state of an oscillator was a function of only its intrinsic heterogeneity (Moon et al., 2006; Laing, 2016). Our expansion (and subsequent truncation of the expansion) in this form is motivated by the large body of work in the field of uncertainty quantification; the difference being that here we have heterogeneous parameters characterizing a single network, rather than many realizations of a dynamical system, each with different (uncertain) parameters. We anticipate that this new link between the two fields (network dynamics and UQ) may provide many more fruitful opportunities for mathematical/computational technology transfer that can enhance our understanding and ability to usefully describe and analyze dynamics on complex networks.

Although, we have only considered Kuramoto-type oscillators in a specific Chung-Lu network, our methods do not rely on either the type of oscillator used or the specific network (as long as the mean degree is not small). Thus, they should be widely applicable to many non-trivial networks of neurons which exhibit synchrony for some range of parameters.

Using this reduced description of a network, we demonstrated a number of standard computational tasks using the equation-free framework, in which differential equations describing the evolution of the expansion coefficients are not explicitly derived, but rather estimated on-the-fly. Specifically, we demonstrated coarse projective integration, the computation of coarse fixed points and their stability, as well as parametric analysis through continuation.

The success of our method relied on the rapid development of correlations between the state of an oscillator and its heterogeneous identifying parameters, in this case, its intrinsic frequency and its degree. A potential shortcoming of the method would arise when such a strong dependence does not develop—that is, when “similar” oscillators do not behave “similarly” (e.g., when the initial conditions, or something more than just the degree, like the clustering coefficient of every node, matters). This implies that additional “heterogeneity dimensions” must be introduced, in analogy to when, say, a two-dimensional flow loses stability and becomes three-dimensional. One such case we have encountered (Moon et al., 2015) is when the oscillators in a network (an all-to-all network of Hodgkin-Huxley neurons) split in two subsets and in each subset a distinct relation of state to identity was established. Knowing the identity of the oscillator was not enough, in that case, to characterize dynamics; one also needed to know in which cluster the oscillator belonged. We could regard both our case as well as this other one as special cases where, for every oscillator identity, there is a distribution of behaviors—a strongly peaked unimodal distribution in this paper, and a strongly peaked bimodal distribution in Moon et al. (2015); for that matter, in our study of breakup in multiple communities/clusters, one obtained a multimodal distribution. We anticipate that, in the spirit of stochastic PDEs in physical space, our approach might be extended to evolve state distributions in heterogeneity space (as opposed to state functions in heterogeneity space).

In other networks, it may be that the state of a node depends on more than just these two properties: networks with weighted edges provide an obvious context in which this may occur. We believe (and are actively pursuing this research direction) that the approach introduced here can also be usefully extended to help in determining reduced descriptions for such networks.

The tensor product basis used here relied on a lack of correlation across the heterogeneities. As mentioned in Section 3.1, this reliance can be overcome by generating the full multidimensional basis all at once, and our current work addresses this possibility. This is likely to be the case in dynamical systems in which it is is useful to retain multiple structural heterogeneities. Degree is one of several structural parameters—others include a node's participation in motifs like triangles (complete graphs on three nodes), cherries (triangles with one edge removed), or its local clustering coefficient. This progression can be continued to higher-order statistics of the node connectivity by noting that using the degree of each node as the representative structural heterogeneity is equivalent to considering the per node counts of the two-node one-edge motif. As more structural heterogeneities are considered, it is reasonable to expect that these heterogeneities will not be statistically independent.

## Author contributions

TB, IK, CL, and CG planned the work, and TB and YW performed the computations. All the authors contributed to the writing of the manuscript.

### Conflict of interest statement

The authors declare that the research was conducted in the absence of any commercial or financial relationships that could be construed as a potential conflict of interest.

## Figures and Tables

**Figure A1:**
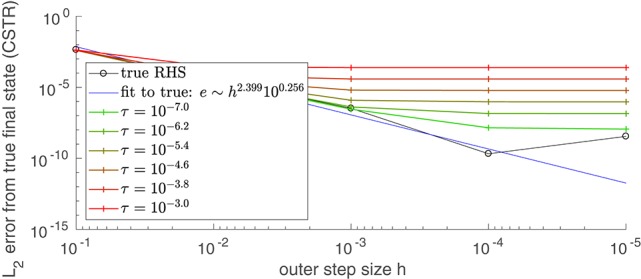
As the inner step size τ decreases, the error (compared to direct integration) of a *projective* integration becomes bounded instead by the integrator's intrinsic (outer) step size *h*. Projective integration is performed on a system of two ODEs modeling a single reversible reaction in a continuously-stirred tank reactor with in- and out-flow. Similar to Figure 5, we quantify the difference between direct integration and projective integration, for various values of τ and *h*. The true solution at *t* = 0.417 was found by integrating using MATLAB's ode45 with an absolute tolerance of 10^−12.0^ and a relative tolerance of 10^−12.0^ The series of black circles give the error at *t* = 0.417 that results from using integration using the true RHS function (Equation 3) in an explicit second-order Runge-Kutta integration scheme of (outer) step size *h*. The colored curves use the same integrator and outer step size, but approximate the RHS function with a difference map analogous to the coarse difference map of Equation (18). Error is evaluated by taking the 2-norm of the vector difference between the projective integration solution *x*(0.417) and the true solution.

## Appendix

### A.1. Orthogonality of the tensor product basis for independent weightings

Here, we demonstrate that if we have polynomials in two variables that are orthogonal with respect to a two-dimensional weight function that is the product of two one-dimensional weight functions, then the orthogonal polynomials are the product of the respective one-dimensional orthogonal sets.

We suppose that we are given two one-dimensional orthogonal function sets defined on (possibly infinite) intervals *I*_*x*_ and *I*_*y*_ based on the weight functions ρ(*x*), *x* ∈ *I*_*x*_, and σ(*y*), *y* ∈ *I*_*y*_ and we want to construct a set of two-variable orthonormal polynomials, ψ_*i,j*_(*x, y*) of degrees *i* in *x* and *j* in *y* such that

(A1) $$\begin{array}{l} {\langle \psi_{i,j},\psi_{m,n}\rangle }_{\rho \sigma }=\delta_{im}\delta_{jn} \end{array}$$

where

(A2) $$\begin{array}{l} {\langle f,g\rangle }_{\rho \sigma }\overset{\text{def}}{=}\iint f(x,y)g(x,y)\rho (x)\sigma (y)\text{d}x\text{d}y \end{array}$$

and {ϕ_*i*_(*x*)} and {θ_*j*_(*y*)} are the orthonormal sets of polynomials corresponding to the one-dimensional systems based on the weights ρ(*x*) and σ(*y*) respectively, that is,

$$\begin{array}{l} {\langle \phi_{i},\phi_{j}\rangle }_{\rho }=\delta_{ij} \end{array}$$

and

$$\begin{array}{l} {\langle \theta_{i},\theta_{j}\rangle }_{\sigma }=\delta_{ij}. \end{array}$$

We will see that these restrictions lead to the product polynomials ψ_*i,j*_(*x, y*) = ϕ_*i*_(*x*)θ_*j*_(*y*).

Since ψ_*i,j*_(*x, y*) has maximum degree of *i* in *x* and *j* in *y*, it can be written as

(A3) $$\begin{array}{l} \psi_{i,j}(x,y)=\sum_{p\leq i,q\leq j}A_{ijpq}\phi_{p}(x)\theta_{q}(y) \end{array}$$

Substituting Equation (A3) in Equation (A1) we get

$$\begin{array}{l} \delta_{im}\delta_{jn}=\sum_{p\leq i,q\leq j}\sum_{r\leq m,s\leq n}A_{ijpq}A_{mnrs}{\langle \phi_{p}(x),\phi_{r}(x)\rangle }_{\rho }\\ \;{\langle \theta_{q}(y),\theta_{s}(y)\rangle }_{\sigma }\\ \;=\sum_{p\leq i,q\leq j}\sum_{r\leq m,s\leq n}A_{ijpq}A_{mnrs}\delta_{pr}\delta_{qs}\\ \;=\sum_{p\leq \min(i,m),q\leq \min(j,n)}A_{ijpq}A_{mnpq} \end{array}$$

We prove the result by induction on *k* = *m*+*n* showing that

$$\begin{array}{l} A_{ijmn}=\delta_{im}\delta_{jn}. \end{array}$$

For *k* = 0 from Equation (B1), we have immediately that $A_{0000}^{2}\;=\;1$ and *A*_*ij*00_*A*_0000_ = 0 for *i* + *j* > 0. Since the signs of the one-dimensional orthogonal polynomials are arbitrary, we can take *A*_0000_ = 1. The second relations implies that *A*_*ij*00_ = 0 for *i* + *j* > 0. This establishes the result for *k* = 0.

Assuming that it is true for *k* − 1, we examine Equation (B1) with *k* = *m* + *n*. Setting *i* = *m* and *j* = *n* in Equation (B1), we have $A_{mnmn}^{2}=1$ allowing us to choose *A*_*mnmn*_ = 1. Then, for any (*i, j*) ≠ (*m, n*) we have *A*_*ijmn*_*A*_*mnmn*_ = 0 implying that *A*_*ijmn*_ = 0, thus establishing the result.

### B.1. Accuracy of integrators using a chord slope rather than the true derivative.

In this paper, we used fine-scale simulation to estimate the derivatives of the coarse variables from their chord slopes. We then used the chord slope in various numerical tasks such as finding steady states and integration. In this Appendix, we address the impact of the errors due to using chord slopes on integrators. Note, that this is unrelated to any errors due to switching back and forth between fine and coarse variables which must be analyzed separately.

We have used both fixed step-size methods and off-the-shelf codes that automatically pick the step size to control an error estimate. We show below the following two lemmas that are valid for Runge-Kutta (RK) and multi-step methods (and probably valid for other classes of methods, but each needs to be examined individually): (1) When a fixed step-size method of order *p* is used, if the chord is of length τ = O(*h*^*p*^) then the resulting method continues to be order *p*; (2) When an automatic method is used, if the error in the chord estimate of the derivative is bounded by the error control amount, then the one-step error of the resulting method is generally increased We discuss these results for the equation ẏ = *f*(*y*).

#### B.1.1. Fixed step-size methods

Any method—whether RK or multi-step—advances from one step to the next by computing an approximation to *y*(*t*+*h*)−*y*(*t*) from a linear combination of approximations to the derivatives at various points—all multiplied by *h* (and also a linear combination of past values in the case of multi-step methods). If the method has order *p* it means that the approximation to the change from *t* to *t* + *h* has an error of O(*h*^*p*+1^). Using a chord slope approximation to a derivative over distance τ gives an error in the derivative estimate of O(τ), so the error in the integration formula is O(τ*h*). Hence, if τ = O(*h*^*p*^) then the additional error is O(*h*^*p*+1^), so the formula remains of order *p*.

If the method handles stiff equations and hence needs a Jacobian to solve the non-linear implicit equation, as long as the Jacobian is calculated numerically (which is the case in most automatic codes), this result does not change, as now the non-linear equation to be solved involves the chord slope rather than the derivative.

#### B.1.2. Automatic methods

Automatic method adjust the step size (and possibly also the order) from step to step to control an error estimate. In the early days of automatic integrators, there was a lot of discussion about whether to control the error per step, or the error per unit step. In error-per-unit-step, the error estimate for a step is controlled to be less than *hϵ* where *h* was the step size used and ϵ was the desired error. The reasoning behind this approach was that, if the errors accumulated more or less linearly from step to step (that depends very much on the stability or otherwise of the differential equation) then the error at the integration end point should be roughly proportional to the integration interval times ϵ. However, if the system is stiff, errors are strongly damped from step to step, so the dominant error is the error in the last step, around ϵ. Many automatic codes, having gone to the effort to estimate the error in a step, correct the solution by the error estimate (although there is now no direct estimate of the higher-order error, an assumption that higher derivatives behave similarly to lower derivatives can provide some comfort). An interesting aspect of this process is that if the original control was on error per step, the error control now becomes per-unit-step because of the increased order.

Regardless of the actual control mechanism used, all methods add a linear combination of derivative estimates multiplied by *h* (and possibly a combination of past solution values) so that the influence of the slope estimates is of the form $h\overset{k}{\underset{i=0}{\sum }}\beta_{i}s_{i}$. If each *s*_*i*_ is controlled to have an error of no greater than ϵ, the contribution to the error is no worse than *Ahϵ*, where $A=\overset{k}{\underset{i=0}{\sum }}|\beta_{i}|$. Thus, the one step error is no worse than *A* + 1 larger than before.

Most automatic codes provide both a relative error tolerance and an absolute error tolerance. Generally we are interested in errors relative to the size of the solution. However, when the solution is almost zero it may be impossible to get an error which is small relative to the solution, so the reason for an absolute error control is to defuse that problem. If either relative or absolute error is bounded, the step proceeds.

We demonstrate the convergence of error as *h* is decreased for various τ on a two-ODE continuously-stirred tank reactor (CSTR) problem with in- and out-flow in Figure A1. “Full” integration is by ode45 with the default tolerances, and the projective integration is a second-order Runge-Kutta scheme. For this example, we model the reaction

(B1) $$\begin{array}{l} \;A\overset{a}{\underset{b}{⇌}}B\\ \frac{\text{d}}{\text{d}t}x=J\cdot x+V\left[\begin{array}{c} x_{A,\text{in}}\\ x_{B,\text{in}} \end{array}\right], \end{array}$$

where $x=\left[\begin{array}{c} x_{A}\\ x_{B} \end{array}\right]$ is the system state, and $\text{J}=\left[\begin{array}{cc} -a-V & b\\ a & -b-V \end{array}\right]$ is the Jacobian. The system parameters are the reaction rates *a* = 2 and *b* = 1, the input flow rate *V* = 0.4, and the input concentrations *x*_*A*, in_ = 1 and *x*_*B*, in_ = 0. The eigenvalues of **J** are λ_1_ = −(*a* + *b* + *V*) and λ_2_ = −*V*. We first take the initial condition to be $x=\left[\begin{array}{c} 1\\ 0 \end{array}\right]$, integrate until *t* = −1/λ_1_/2, half of the first timescale. We use this state as the common initial condition for the integrators in Figure A1. As can be seen, the error of the method decreases with the inner step size τ until a bound governed by the outer step size *h* is reached. As predicted by the analysis above, this governing function is close to the curve O(h^p^).
